# A natural PKM2 targeting agent as a potential drug for breast cancer treatment

**DOI:** 10.1002/ctm2.1157

**Published:** 2022-12-28

**Authors:** Xin‐Yue Shang, Yu‐Jue Wang, Zi‐Lin Hou, Xin‐Ye Wang, Hao Zhang, Chen‐Yu Yang, Ji‐Chong Li, Xiao‐Xiao Huang, Shao‐Jiang Song, Guo‐Dong Yao

**Affiliations:** ^1^ Key Laboratory of Computational Chemistry‐Based Natural Antitumor Drug Research & Development Engineering Research Center of Natural Medicine Active Molecule Research & Development Shenyang China; ^2^ Key Laboratory of Natural Bioactive Compounds Discovery & Modification Shenyang Liaoning China; ^3^ Department of Natural Medicine Chemistry, School of Traditional Chinese Materia Medica Shenyang Pharmaceutical University Shenyang Liaoning China; ^4^ Department of Pharmacology Shenyang Medical College Shenyang Liaoning China

Dear editor,

Breast cancer was the most common cancer and had the highest mortality rate in women worldwide.^1^, ^2^, ^3^, ^4^ Yuanhuacine (YHC), a daphnane‐type diterpenoid as the main active ingredients, inhibited breast cancer cell growth, but the detailed mechanism had not yet been described.^5^, ^6^ This study aimed to investigate the target of YHC that induced breast cancer cells death and explored the underlying mechanisms.

Our previous studies showed that YHC exhibited significant inhibitory activity against various human tumour cell lines, especially breast cancer cells.^5^ Thus, we tested the cytotoxicity of YHC against MCF‐7, MDA‐MB‐361 and BT549 breast cancer cells in vitro by MTT assay with Tamoxifen (Tam) and Doxorubicin (Dox) as the positive controls (Table S1). The results showed a concentration‐dependent decrease in the viability of breast cancer cells after treatment with YHC (Figure S1A–C). Moreover, YHC was less toxic than positive controls (Dox and Tam) on human normal breast epithelial cells MCF‐10A cells (Figure S2).

Xenograft models were established for evaluating the anti‐tumour effect of YHC (Figure 1A). YHC significantly inhibited the growth of xenograft tumours (Figures 1B–D). The mice's body weight was not affected by YHC treatment (Figure 1E), indicating that the dosage of the treatment was not overtly toxic. Additionally, each organ was not notably different among the YHC‐treated groups and the control groups (Figures 1F–K，Table S2). Furthermore, tumour tissues from the YHC group revealed a lower level of Ki‐67 expression by immunohistochemistry (Figure 1L).

**FIGURE 1:**
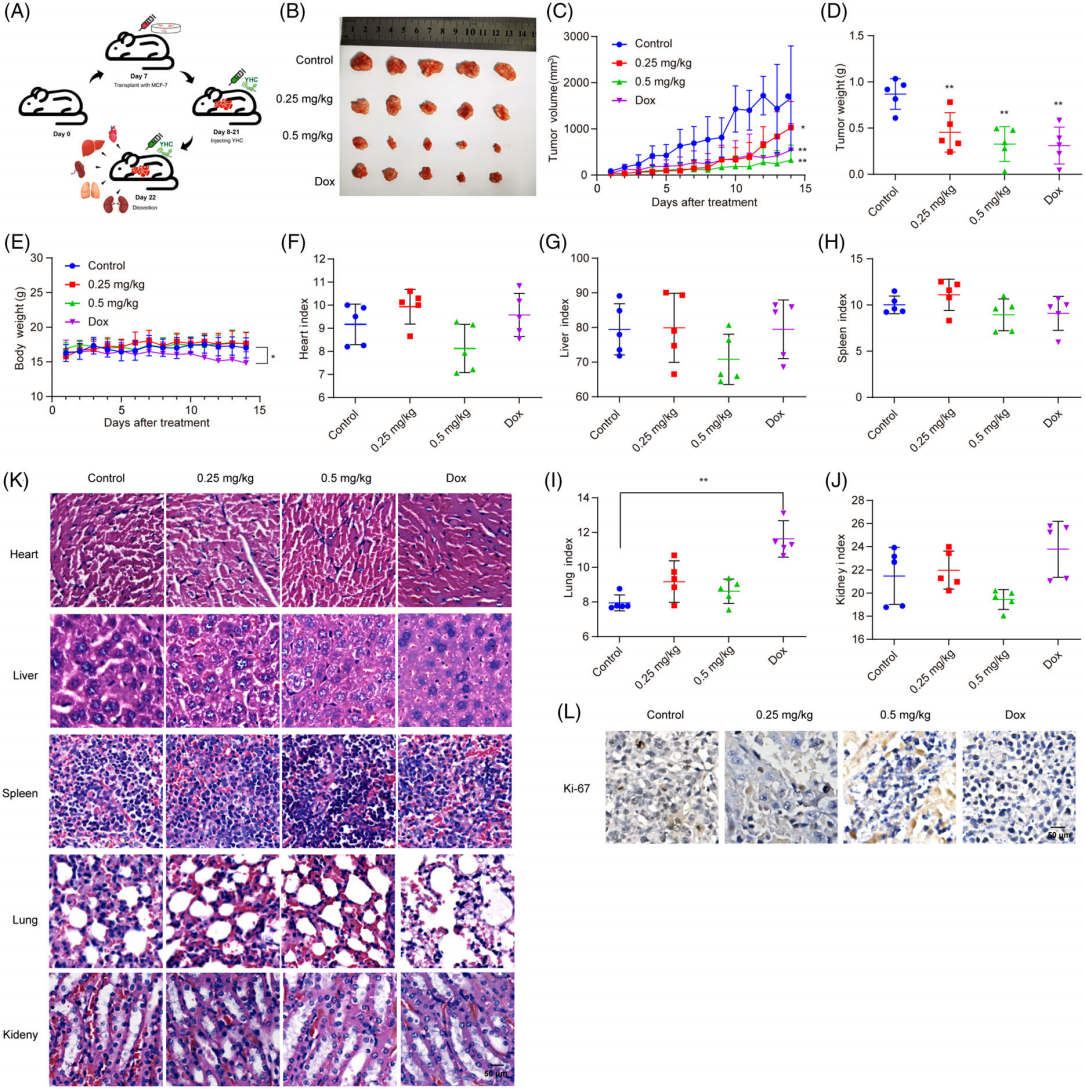
The in vivo anti‐tumour effect of YHC on human breast cancer MCF‐7 cells by using xenograft models. (A) The experimental flow chart for evaluating the anti‐tumour effect of YHC. (B) The tumour issues were excised and photographed after treatment with YHC for 14 days. (C) Tumour volumes were measured using calipres for each group every day. (D) Tumour weights were detected in each group. (E) The body weights of animals were recorded for each group during 14 days. (F–J) The organ index of nude mice was calculated for heart (F), liver (G), spleen (H), lung (I) and kidney (I), respectively. (K) Representative histomorphological changes of the organs were assessed after H&E staining. (L) YHC could decrease Ki‐67 expression in tumour tissues by immunohistochemistry assay. Statistical analysis was performed on the data as the mean ± SD (*n* = 5). ^*^ *p* < .05, ^**^ *p* < .01 versus control group.

Compared with the control group, YHC‐treated cells exhibited nuclear condensation and fragmentation, which were hallmarks of apoptotic cells (Figure S3A). Next, apoptosis in breast cancer cells was observed after YHC was stained with Annexin V‐FITC/PI and Western blot (Figures S3B–E).

DARTS/MS method had a powerful advantage identifying targets of natural products.^7^, ^8^ YHC treatment‐enriched bands occurred at molecular weights of approximately 55–72 kDa (Figure S4A). A total of 1007 proteins within the target molecular weight range were tested in MCF‐7 cells (Figure S4B). A peak corresponding to pyruvate kinase M2 (PKM2) was identified by mass spectrometry, and it was one of the proteins with the greatest significant difference (Figures S4C and D). Moreover, the functions and mechanisms of the changed proteins were evaluated by Kyoto encyclopaedia of gene and genomes, gene ontology and protein–protein interaction (PPI) networks, which indicated that YHC mainly regulated the citrate cycle (TCA cycle) and pyruvate metabolism (Figures S4E–G). Breast cancer patients had high expression of the PKM2 gene (Figure S4H). According to the survival rate of patients, breast cancer patients born high PKM2 level had a poor prognosis (Figure S4I). We detected the abundance of PKM2 in breast cancer cell lines, it was shown that they had greater expression than normal breast cells (Figure S4J).

Molecular docking showed that YHC and PKM2 had a good bind affinity (Figure 2A). The presence of YHC made the PKM2 was insensitive to proteolysis (Figures 2B and S5A). The PKM2 protein could still be detected with the increasing temperatures in YHC‐treated group (Figures 2C and S5B). YHC bound PKM2 with a *K*_d_ value of 26.3 μM by surface plasmon resonance (SPR) assay (Figure 2D). Next, the results showed that both mRNA and protein levels of prototype PKM2 did not change significantly after treatment with YHC (Figures 2E and F). However, YHC downregulated the expression of p‐PKM2 (Y105) (Figures 2F and S5C). After transfecting the cells with PKM2 siRNA, PCR and Western blot analysis indicated that the mRNA (Figure 2G) and protein (Figures 2H and S6A) levels of PKM2 were decreased. The results suggested that the downregulation of PKM2 exhibited a significant proapoptotic effect after treatment with YHC (Figures 2I, J and S6B, C). Moreover, the overexpression of PKM2 weakened the regulatory effects of YHC on apoptosis (Figures 2K, L and S6D, E).

**FIGURE 2:**
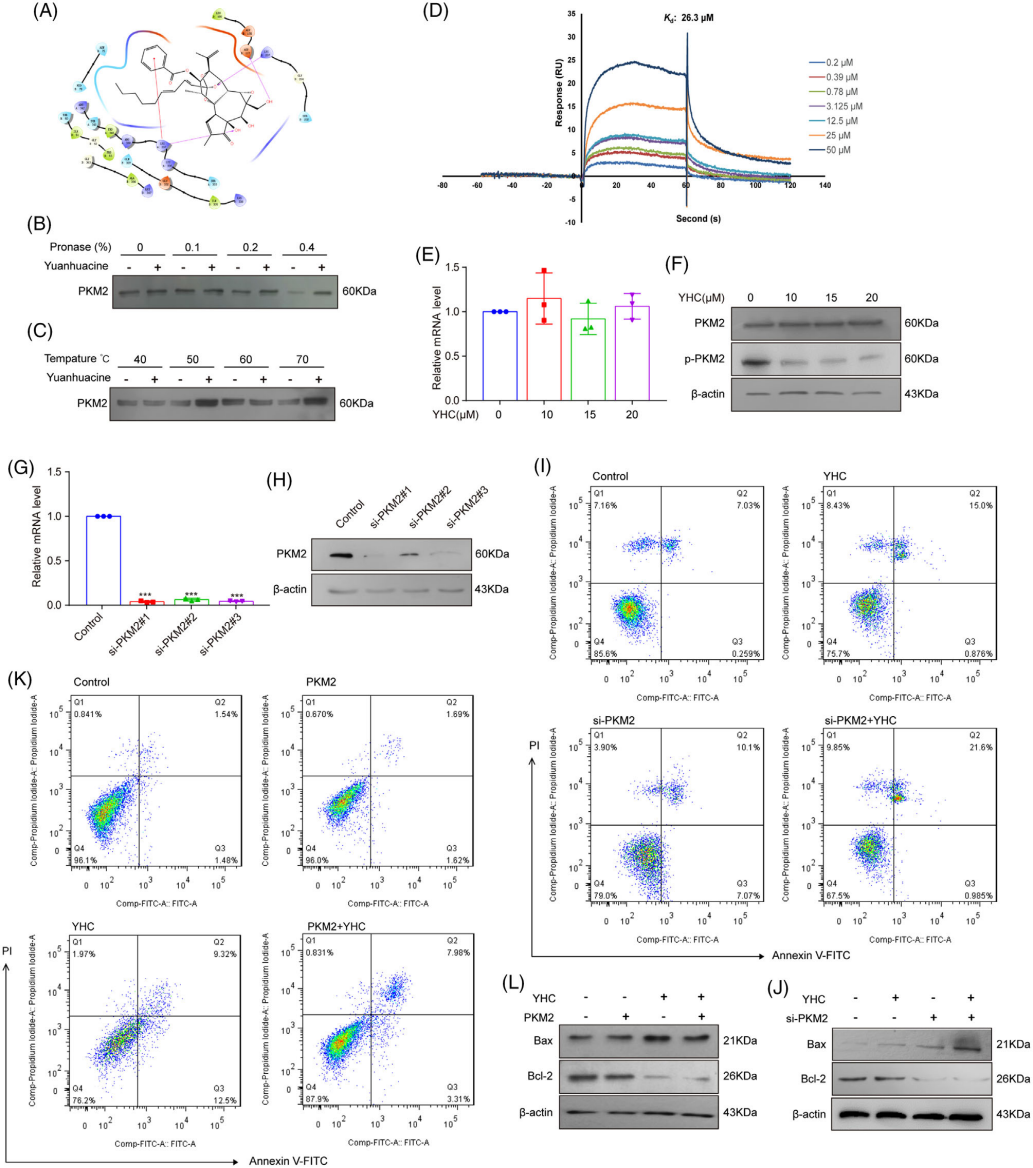
YHC bound with PKM2 directly and promoted breast cancer cells apoptosis. (A) The molecular docking study of YHC and PKM2 (PDB: 6NU5). (B) In the presence or absence of YHC, DARTS assay was used to examine the immunoblot of PKM2 in MCF‐7 cells which were proteolysed with different concentrations of pronase. (C) CETSA assay was applied to detect the stabilisation between YHC and PKM2 at different temperatures. (D) The binding affinity of YHC and PKM2 was examined by SPR assay. (E) RT‐PCR assay was performed for testing the mRNA levels of PKM2 in MCF‐7 cells. (F) The expressions of PKM2 and p‐PKM2 (Y105) were tested by Western blot in MCF‐7 cells. (G and H) The expression of PKM2 after the silencing of PKM2 in MCF‐7 cells by RT‐PCR and Western blot (si‐PKM2#1 was used for following experiments). (I) Apoptotic ratio of YHC treatment in PKM2 siRNA‐treated MCF‐7 cells was tested by Annexin V‐FITC/PI staining. (J) The expression levels of Bax and Bcl‐2 in PKM2 siRNA‐treated MCF‐7 cells by Western blot. (K) Apoptotic ratio of YHC treatment in PKM2‐overexpressed MCF‐7 cells by Annexin V‐FITC/PI staining. (L) The expression levels of Bax and Bcl2 in PKM2‐overexpressed MCF‐7 cells by Western blot. Statistical analysis was performed on the data as the mean ± SD (*n* = 3). ^***^ *p* < .001 versus control group.

The rate‐limiting enzyme PKM2 played an important role in tumour glycolysis.^9^ Thus, after treatment with YHC, breast cancer cells consumed less glucose and produced less lactate (Figure S7A). The related genes (HK2, GLUT1 and LDHA) were all decreased by YHC, as shown by the measurement of their mRNA (Figure S7B) and protein levels (Figures S7C and D).

Recently, the knockdown of PKM2 and STAT3 both increased drug efficacy.^10^ In this study, we found that PKM2 and STAT3 interactions were also inhibited by YHC (Figures 3A and B), suppressed the p‐STAT3 (Y705), CDC2 and Cyclin B1 (Figure 3C). In spite of our investigation of STAT3's potential thermal stability shift caused by YHC, no significant effects were observed (Figure 3D). Furthermore, YHC treatment decreased the expression of p‐PKM2 and p‐STAT3 in vivo, respectively (Figure 3E).

**FIGURE 3:**
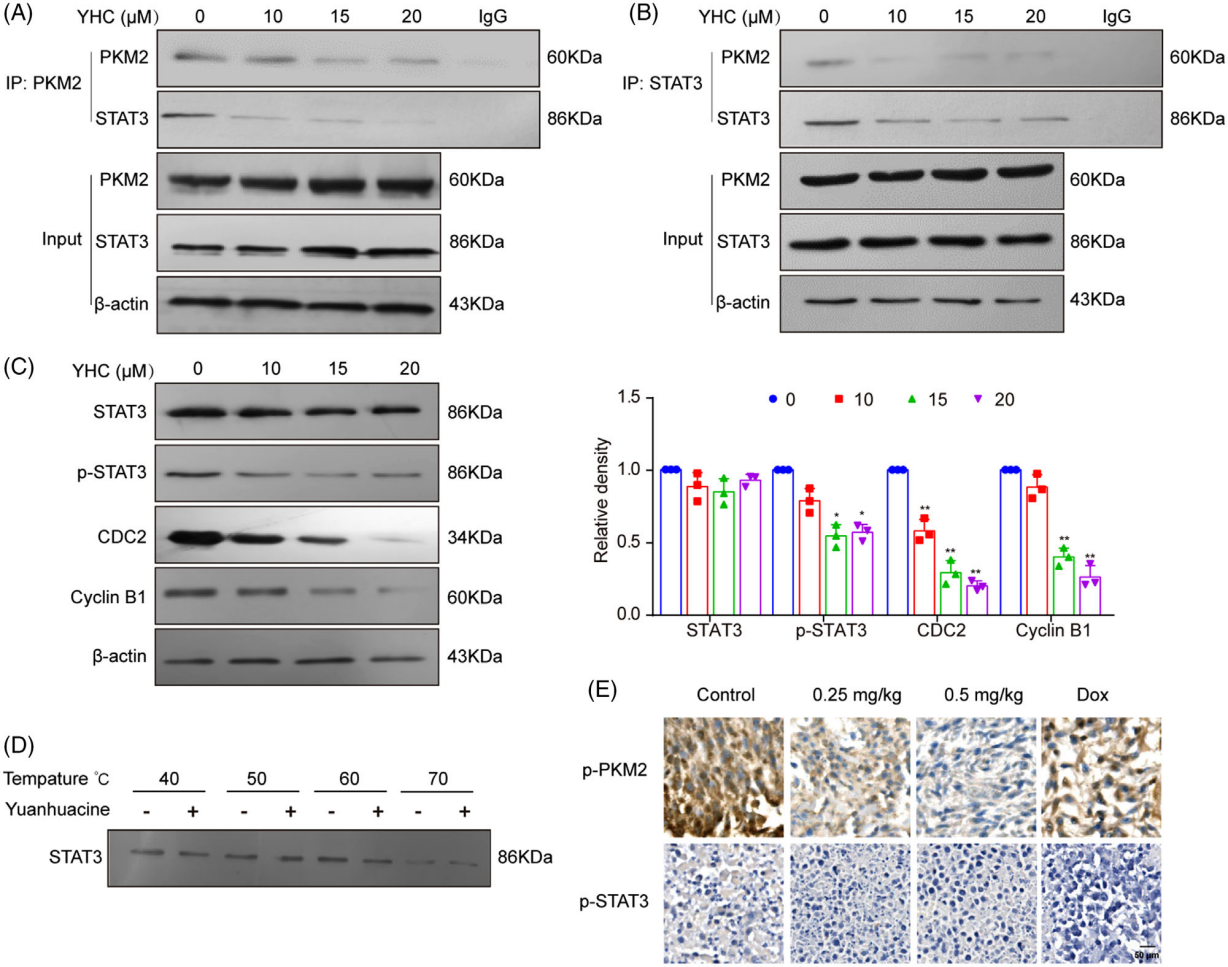
YHC disrupted interactions of PKM2 and STAT3 in breast cancer cells. (A and B) Cells were treated with 0, 10, 15 and 20 μM of YHC for 48 h. The co‐immunoprecipitation assay was used to detect the protein–protein interaction between PKM2 and STAT3. (C) The protein expressions of STAT3, p‐STAT3 (Y705), CDC2 and Cyclin B1 were detected by Western blot assay. (D) CETSA assay was applied to detect the stabilisation between YHC and STAT3 at different temperatures. Statistical analysis was performed on the data as the mean ± SD (*n* = 3). (E) YHC could decrease p‐PKM2 and p‐STAT3 expression in tumour tissues by immunohistochemistry assay. Statistical analysis was performed on the data as the mean ± SD (*n* = 5). ^*^p < .05, ^**^p < .01 versus control group.

STAT3 inhibitor (Stattic) was used to identify the effects of STAT3 pathway. At first, the results showed that combined with Stattic enhanced YHC‐induced apoptosis notably (Figures S8A and B). Subsequently, the combination of Stattic and YHC could significantly inhibit glycolysis (Figures S8C and D). In addition, Stattic enhanced the inhibitory effects of YHC on the expression of glycolysis‐related genes when combined with YHC (Figure S8E). Compared with YHC alone group, p‐STAT3 (Y705) and its downstream could be significantly down‐regulated when Stattic was combined with YHC (Figures S8F and G).

The combination of YHC and PKM2 siRNA treatment markedly inhibited the p‐STAT3 (Y705) and its downstream, compared with YHC treatment alone (Figures S9A and B). YHC‐induced glycolysis was synergistically increased in PKM2 siRNA‐treated cells compared to YHC‐treated cells (Figures S9E–G, K and L). In addition, PKM2 overexpression counteracted the YHC‐inhibited p‐STAT3 (Y705) and its downstream (Figures S9C and D). YHC‐inhibited glycolysis was reversed in PKM2‐overexpressing cells (Figures S9H–J, M and N).

In conclusion, YHC could restrict the growth of breast cancer cells and induced apoptosis in vivo and in vitro. Mechanistically, YHC disrupted interactions of PKM2 and STAT3 to inhibit the downstream proteins. In addition, YHC inhibited breast cancer cells by targeting PKM2 to regulate STAT3 pathway and glycolysis. It not only provided a basis for confirming the target of daphnane‐type diterpenoids, but also enriched the application of natural drug resources in the field of breast cancer therapy.

## AUTHOR CONTRIBUTIONS

Guo‐Dong Yao and Shao‐Jiang Song designed the project. Xin‐Yue Shang performed most of the experiments, analyzed data and wrote the manuscript. Yu‐Jue Wang, and Xin‐Ye Wang performed cell biology experiments and contributed to the manuscript editing. Chen‐Yu Yang performed in vivo experiments. Hao Zhang and Xiao‐Xiao Huang performed extraction and separation of YHC. Ji‐Chong Li performed molecular docking. All authors contributed and approved the final version of the manuscript.

## CONFLICT OF INTEREST

We declare that we have no financial and personal relationships with other people or organisations that can inappropriately influence our work.

## Supporting information

Supporting information

Supporting information

Supporting information

Supporting information

Supporting information

Supporting information

Supporting information

Supporting information

Supporting information

Supporting information

Supporting information

Supporting information

## Data Availability

Data are available upon reasonable request from the corresponding author.
