# Supplementary figures and images for: A natural PKM2 targeting agent as a potential drug for breast cancer treatment
Source: Clin Transl Med. 2022 Dec 28;13(1):e1157. doi: 10.1002/ctm2.1157 (PMC9798039; doi:10.1002/ctm2.1157)

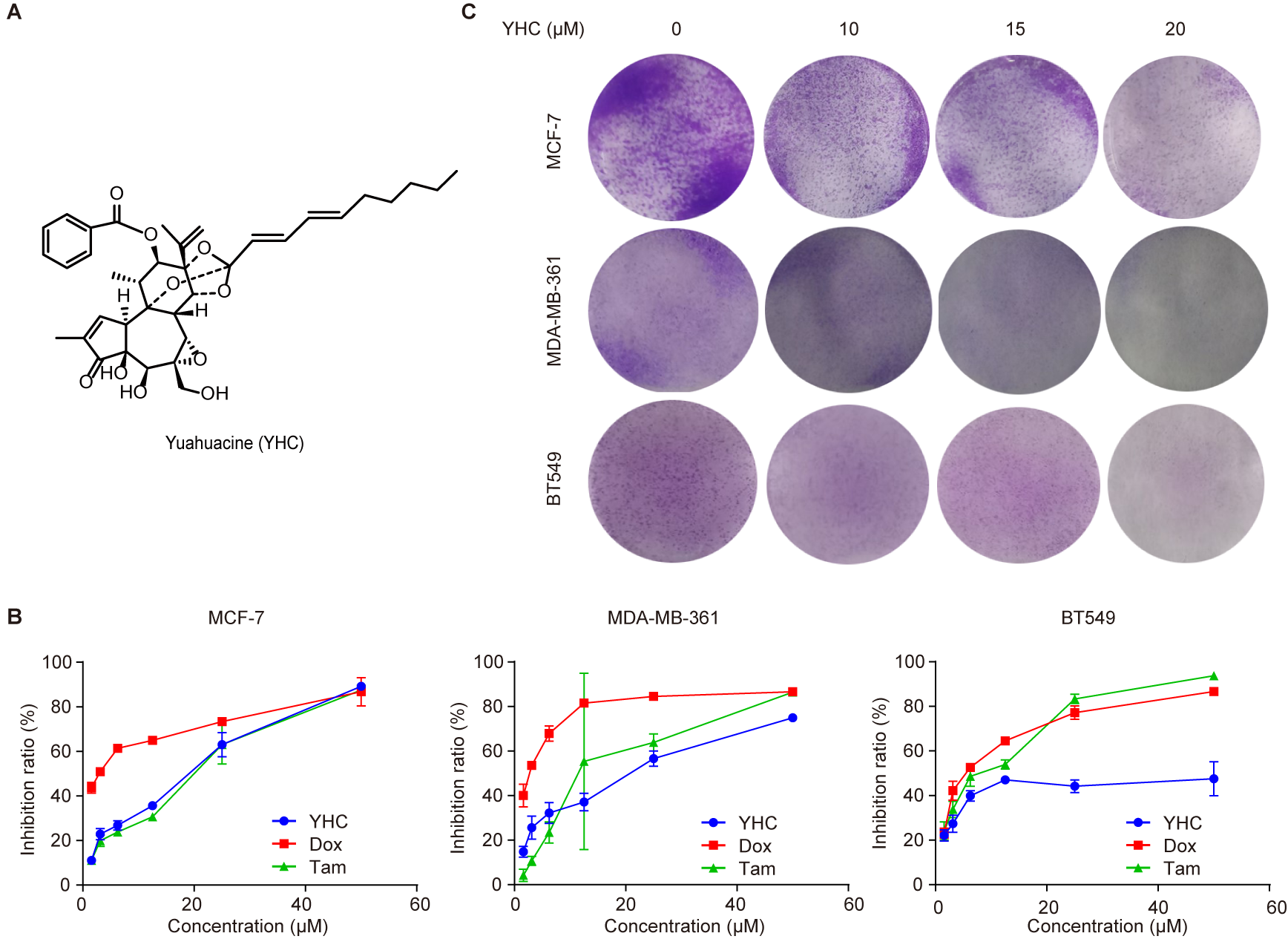

Supplement: Supplementary file 1 — Supporting information [file CTM2-13-e1157-s003.tif]

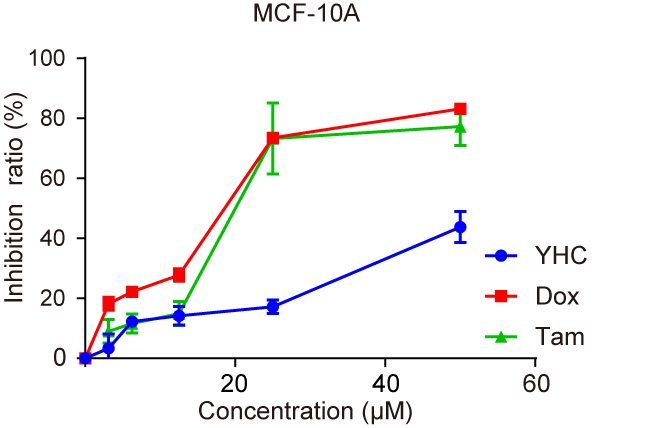

Supplement: Supplementary file 2 — Supporting information [file CTM2-13-e1157-s005.tif]

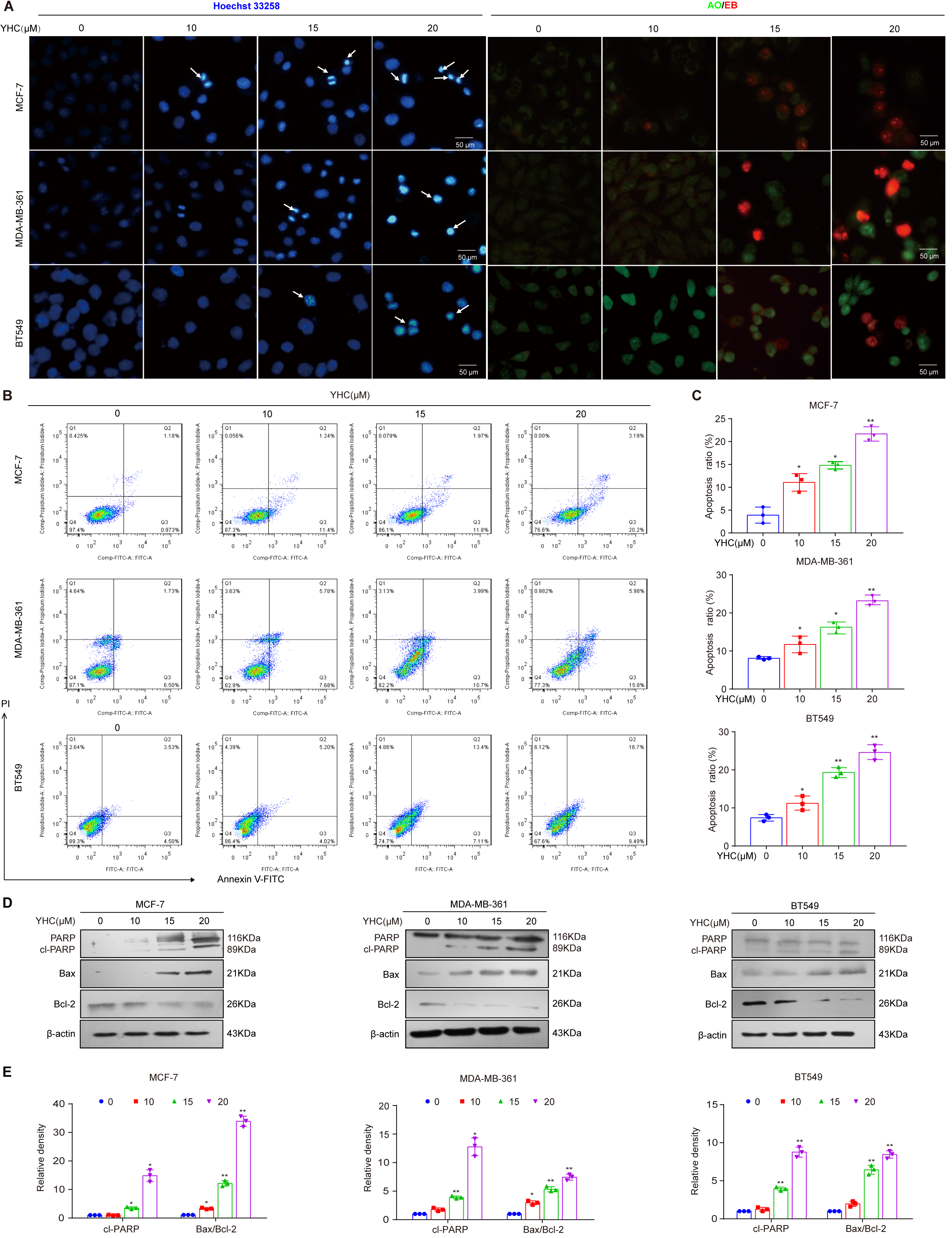

Supplement: Supplementary file 3 — Supporting information [file CTM2-13-e1157-s010.tif]

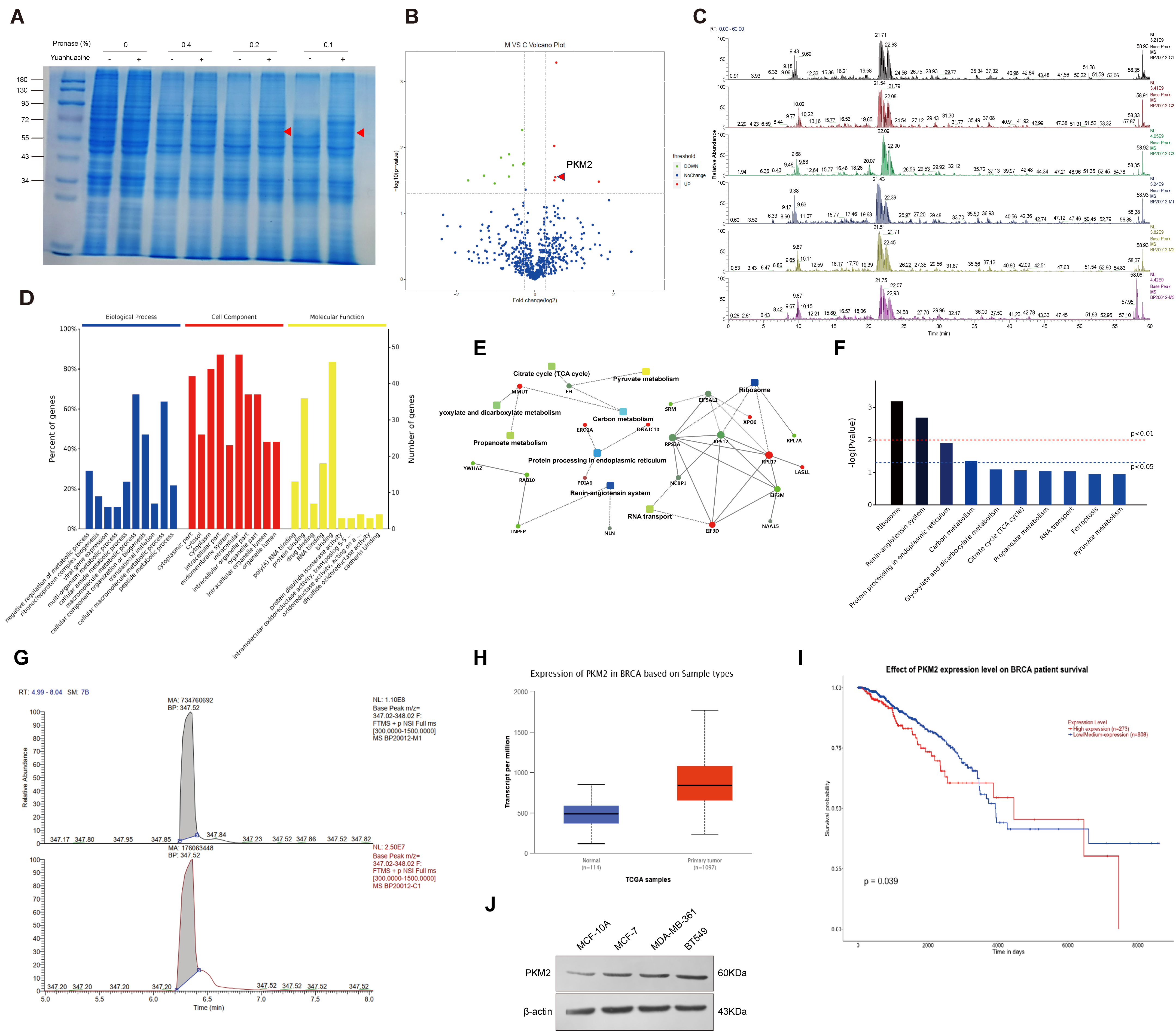

Supplement: Supplementary file 4 — Supporting information [file CTM2-13-e1157-s006.tif]

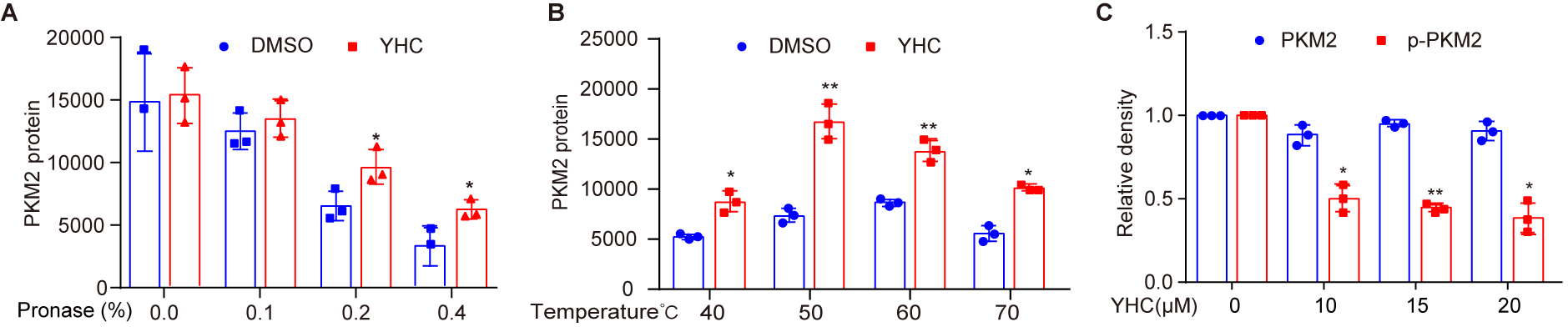

Supplement: Supplementary file 5 — Supporting information [file CTM2-13-e1157-s008.tif]

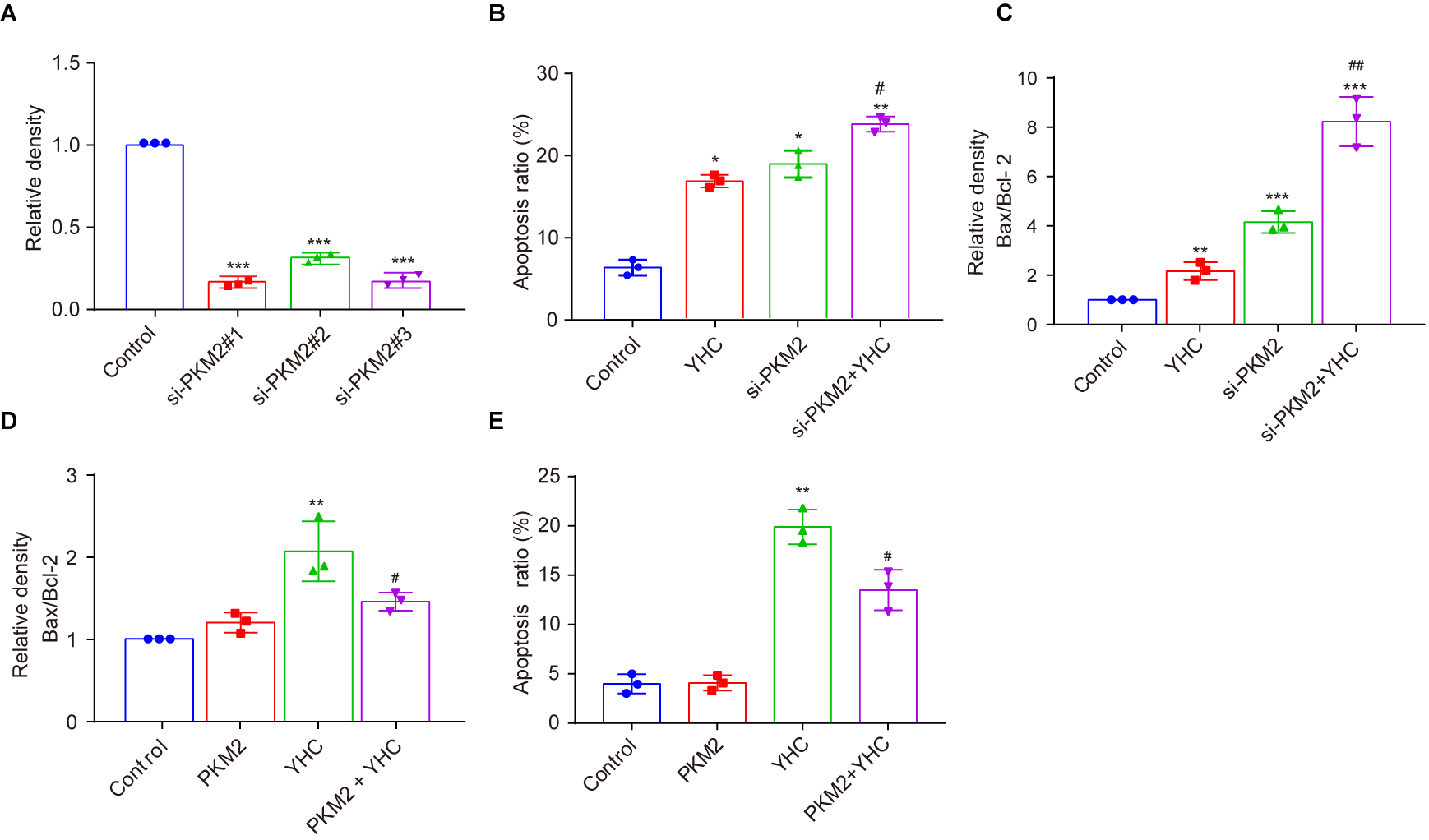

Supplement: Supplementary file 6 — Supporting information [file CTM2-13-e1157-s007.tif]

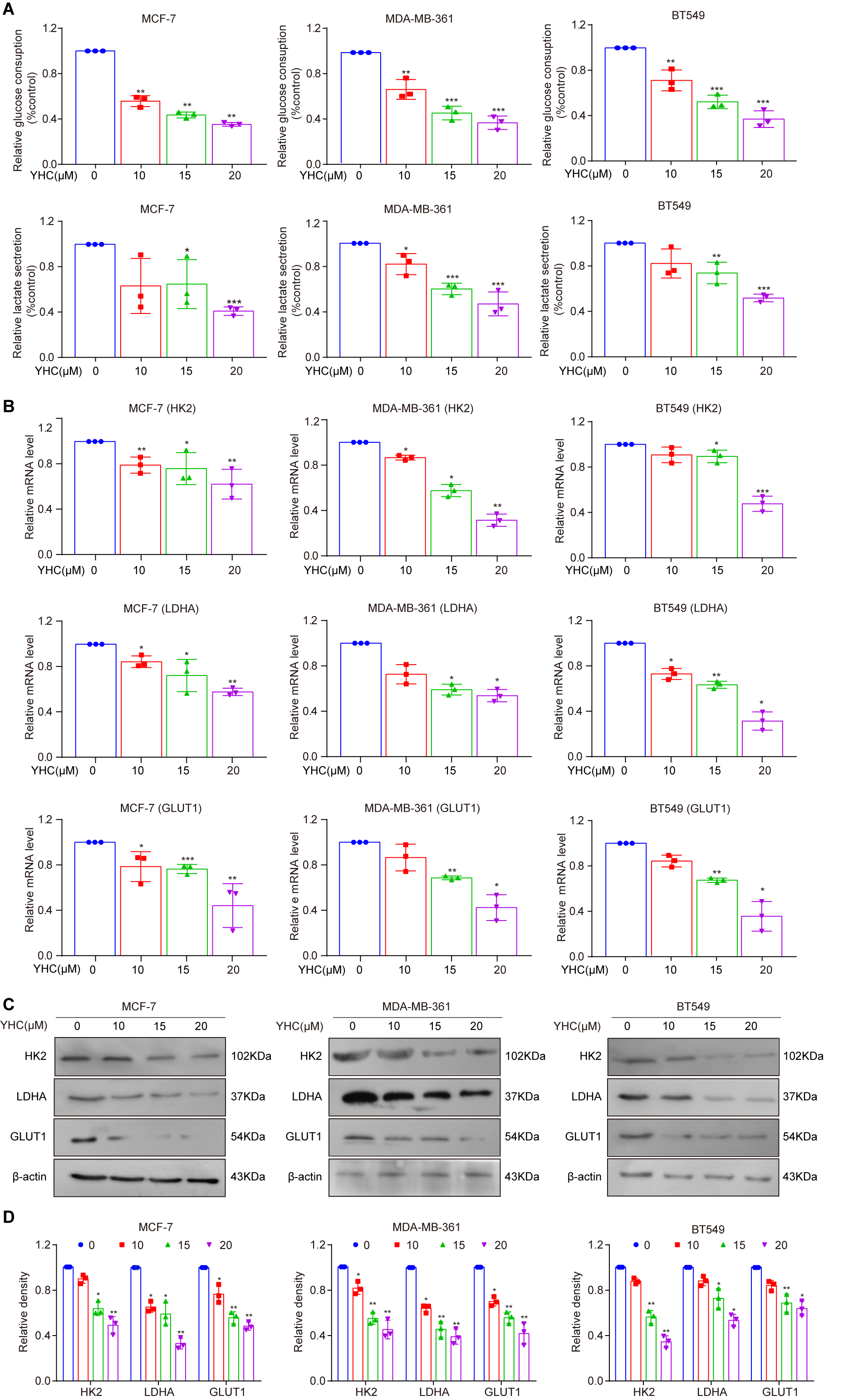

Supplement: Supplementary file 7 — Supporting information [file CTM2-13-e1157-s001.tif]

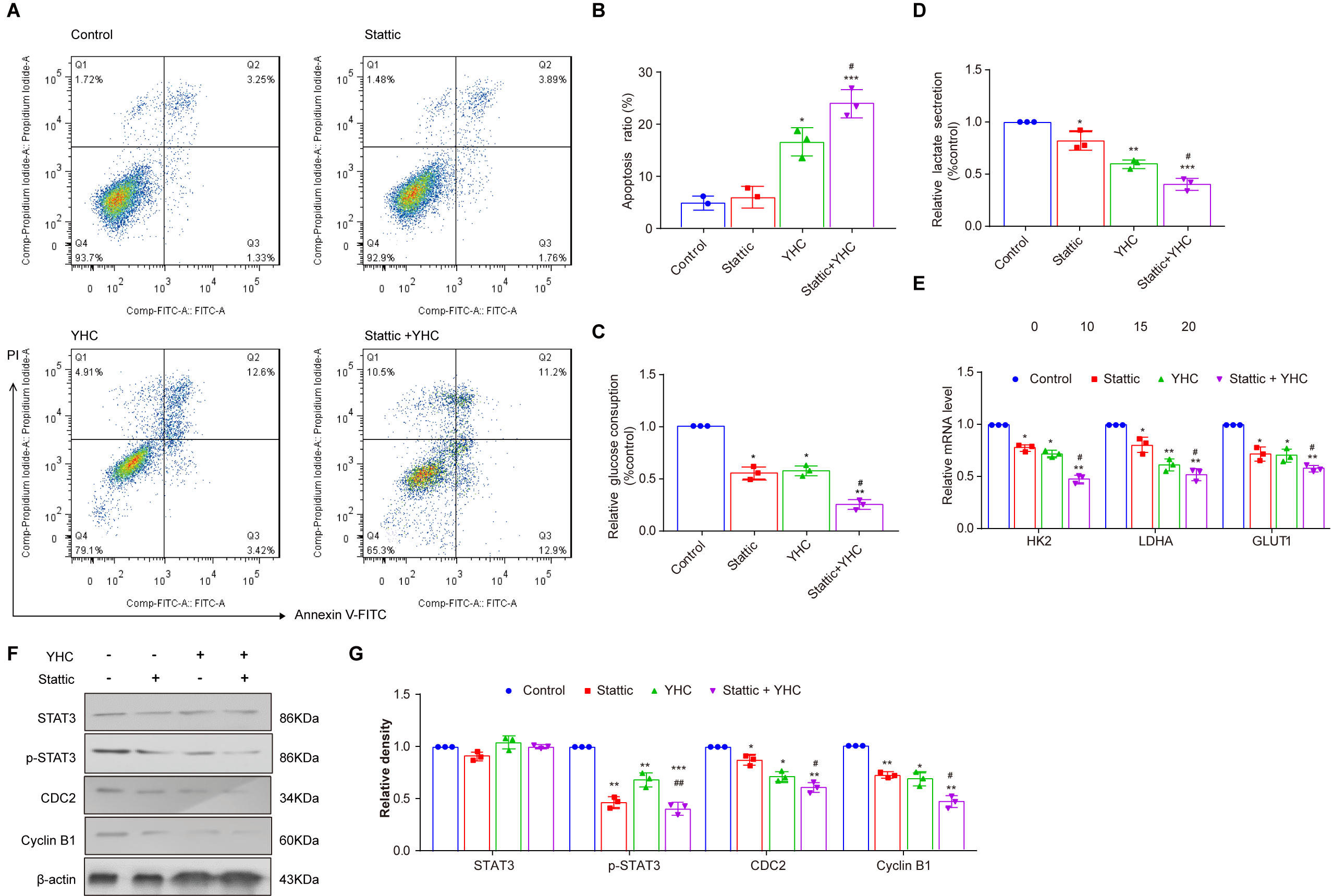

Supplement: Supplementary file 8 — Supporting information [file CTM2-13-e1157-s011.tif]

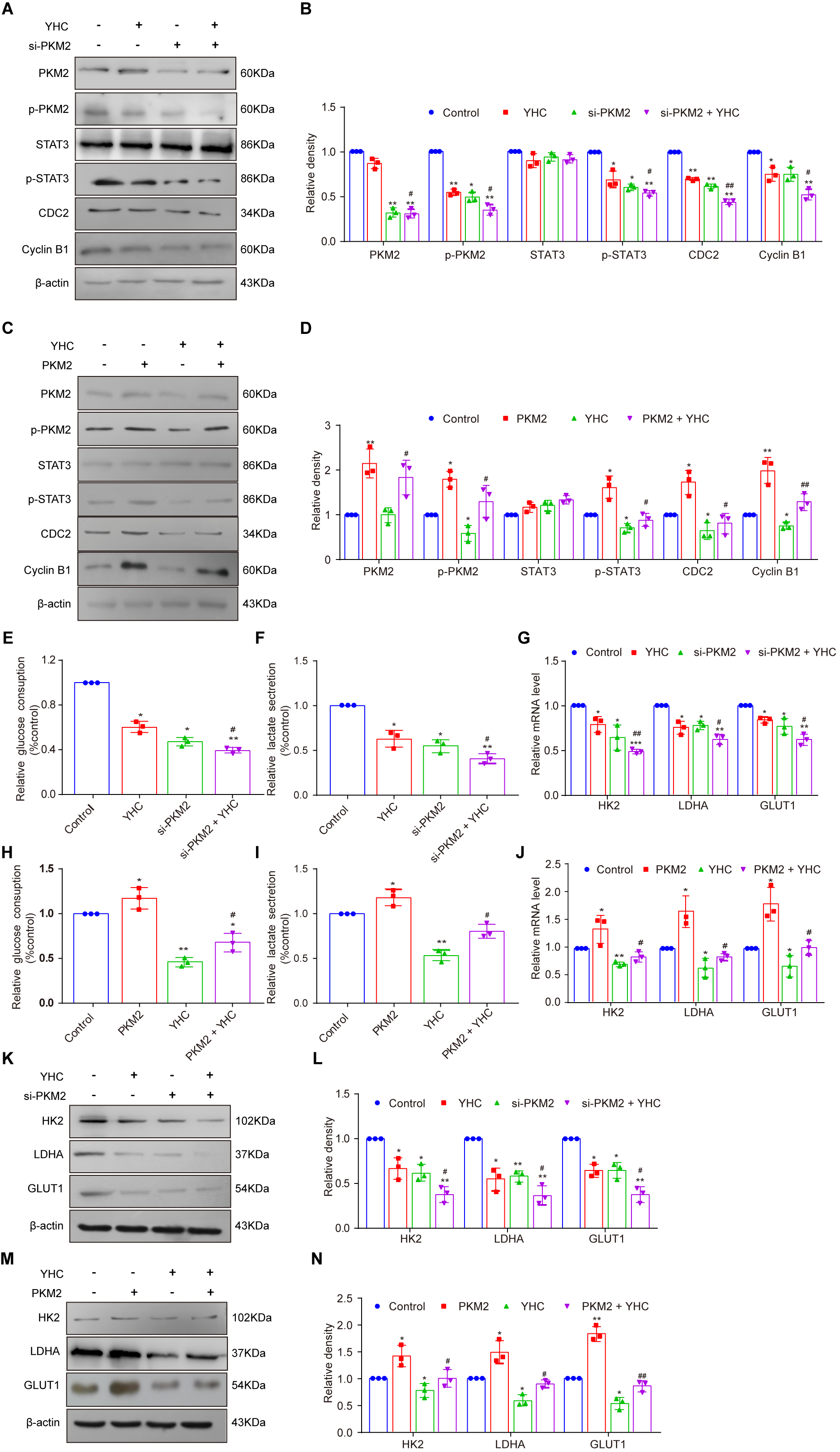

Supplement: Supplementary file 9 — Supporting information [file CTM2-13-e1157-s004.tif]
