# Supplementary material for: A natural PKM2 targeting agent as a potential drug for breast cancer treatment
Source: Clin Transl Med. 2022 Dec 28;13(1):e1157. doi: 10.1002/ctm2.1157 (PMC9798039; doi:10.1002/ctm2.1157)
Supplement: Supplementary file 10 — Supporting information [file CTM2-13-e1157-s002.docx]

**Supporting Information 1:** Supplementary tables

A natural PKM2 targeting agent as a potential drug for breast cancer treatment

Xin-Yue Shang^a, b^, Yu-Jue Wang^a^, Zi-Lin Hou^a^, Xin-Ye Wang^a^, Hao Zhang^a^, Chen-Yu Yang^a^, Ji-Chong Li^a^, Xiao-Xiao Huang^a^, Shao-Jiang Song^a*^, Guo-Dong Yao^a*^

*^a^**Key Laboratory of Computational Chemistry-Based Natural Antitumor Drug Research & Development, Liaoning Province; Engineering Research Center of Natural Medicine Active Molecule Research & Development, Liaoning Province; Key Laboratory of Natural Bioactive Compounds Discovery & Modification, Shenyang; School of Traditional Chinese Materia Medica, Shenyang Pharmaceutical University, Shenyang, Liaoning 110016, China*

*^b^Department of Pharmacology, Shenyang Medical College, Shenyang, Liaoning 110034, China.*

*Correspondence author.

Prof. Shao-Jiang Song, songsj99@163.com;

Prof. Guo-Dong Yao, guodong_yao@126.com;

School of Traditional Chinese Materia Medica, Shenyang Pharmaceutical University, Shenyang, Liaoning Province, People’s Republic of China; Phone: + 86 24 43520793; Fax: + 86 24 43520793

Table S1 The IC_50_ (μM) value of compounds in different cell lines

| Compounds  Cell lines | YHC | Dox | Tam |
| --- | --- | --- | --- |
| MCF-7 | 14.81 ± 3.40 | 2.29 ± 4.78 | 14.43 ± 5.76 |
| MDA-MB-361 | 16.33 ± 5.81 | 2.50 ± 4.12 | 10.62 ± 6.24 |
| BT549 | 18.27 ± 4.79 | 2.52 ± 5.53 | 6.99 ± 4.73 |
| MCF-10A | 71.74 ± 6.82 | 15.88 ± 6.53 | 23.37 ± 5.78 |

Table S2 p-values of each group compared with control group of organ index

| Groups  Organ | 0.25 mg/kg | 0.5 mg/kg | Dox |
| --- | --- | --- | --- |
| Heart | 0.1774 | 0.1264 | 0.4963 |
| Liver | 0.9350 | 0.0986 | 0.9968 |
| Spleen | 0.2467 | 0.2553 | 0.3480 |
| Lung | 0.0506 | 0.1124 | 0.0079 |
| Kidney | 0.7161 | 0.1189 | 0.1734 |
