# Supplementary material for: A natural PKM2 targeting agent as a potential drug for breast cancer treatment
Source: Clin Transl Med. 2022 Dec 28;13(1):e1157. doi: 10.1002/ctm2.1157 (PMC9798039; doi:10.1002/ctm2.1157)
Supplement: Supplementary file 11 — Supporting information [file CTM2-13-e1157-s012.docx]

**Supporting Information 2:** Supplementary Figures

A natural PKM2 targeting agent as a potential drug for breast cancer treatment

Xin-Yue Shang^a, b^, Yu-Jue Wang^a^, Zi-Lin Hou^a^, Xin-Ye Wang^a^, Hao Zhang^a^, Chen-Yu Yang^a^, Ji-Chong Li^a^, Xiao-Xiao Huang^a^, Shao-Jiang Song^a*^, Guo-Dong Yao^a*^

*^a^**Key Laboratory of Computational Chemistry-Based Natural Antitumor Drug Research & Development, Liaoning Province; Engineering Research Center of Natural Medicine Active Molecule Research & Development, Liaoning Province; Key Laboratory of Natural Bioactive Compounds Discovery & Modification, Shenyang; School of Traditional Chinese Materia Medica, Shenyang Pharmaceutical University, Shenyang, Liaoning 110016, China*

*^b^Department of Pharmacology, Shenyang Medical College, Shenyang, Liaoning 110034, China.*

*Correspondence author.

Prof. Shao-Jiang Song, songsj99@163.com;

Prof. Guo-Dong Yao, guodong_yao@126.com;

School of Traditional Chinese Materia Medica, Shenyang Pharmaceutical University, Shenyang, Liaoning Province, People’s Republic of China; Phone: + 86 24 43520793; Fax: + 86 24 43520793


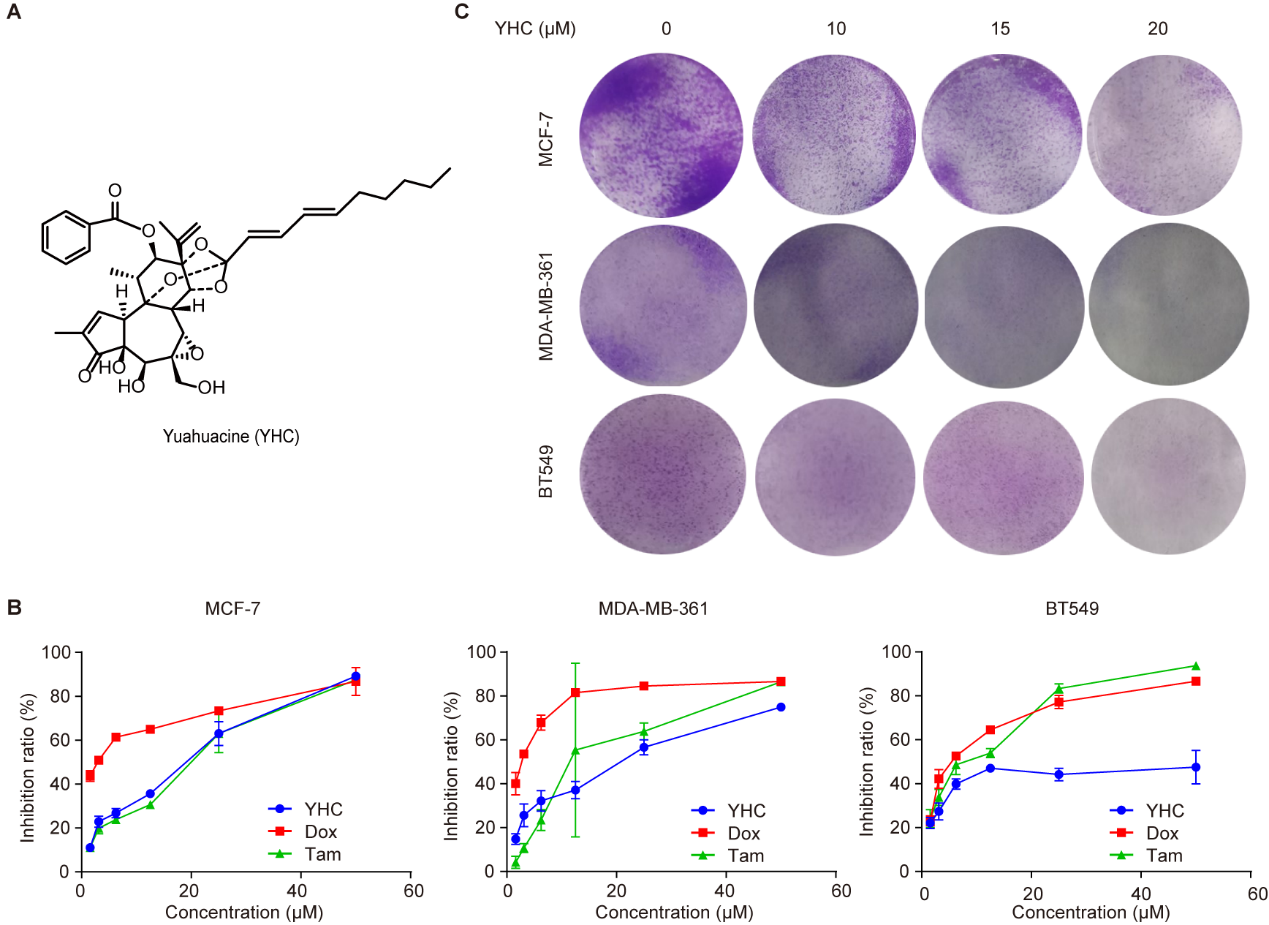


Figure S1 **Effects of YHC on cell viability of human breast cancer cells.**

**A.** The chemical structure of YHC. **B.** The cell growth inhibition curves of YHC, Dox and Tam (0-50 μM) against MCF-7, MDA-MB-361 and BT549 cells for 48 h were detected by the MTT assay. **C.** Colony formation assay of clones in MCF-7, MDA-MB-361 and BT549 cells with treatment of different concentrations YHC (0, 10, 15, 20 μM) for 12 days. The data were presented as the mean ± SD (n = 3).


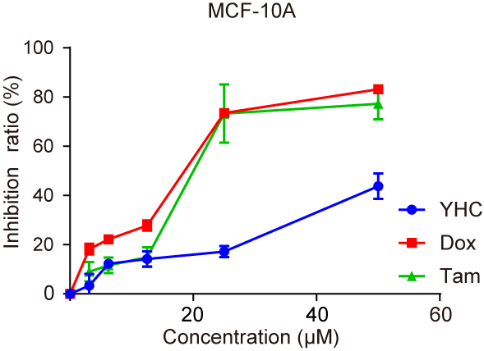


Figure S2 **The cytotoxic effect of YHC on human normal breast cells.**

The cell growth inhibition curves of YHC, Dox and Tam (0-50 μM) in human normal human breast epithelial MCF-10A cells were detected by the MTT assay. The data were presented as the mean ± SD (n = 3).


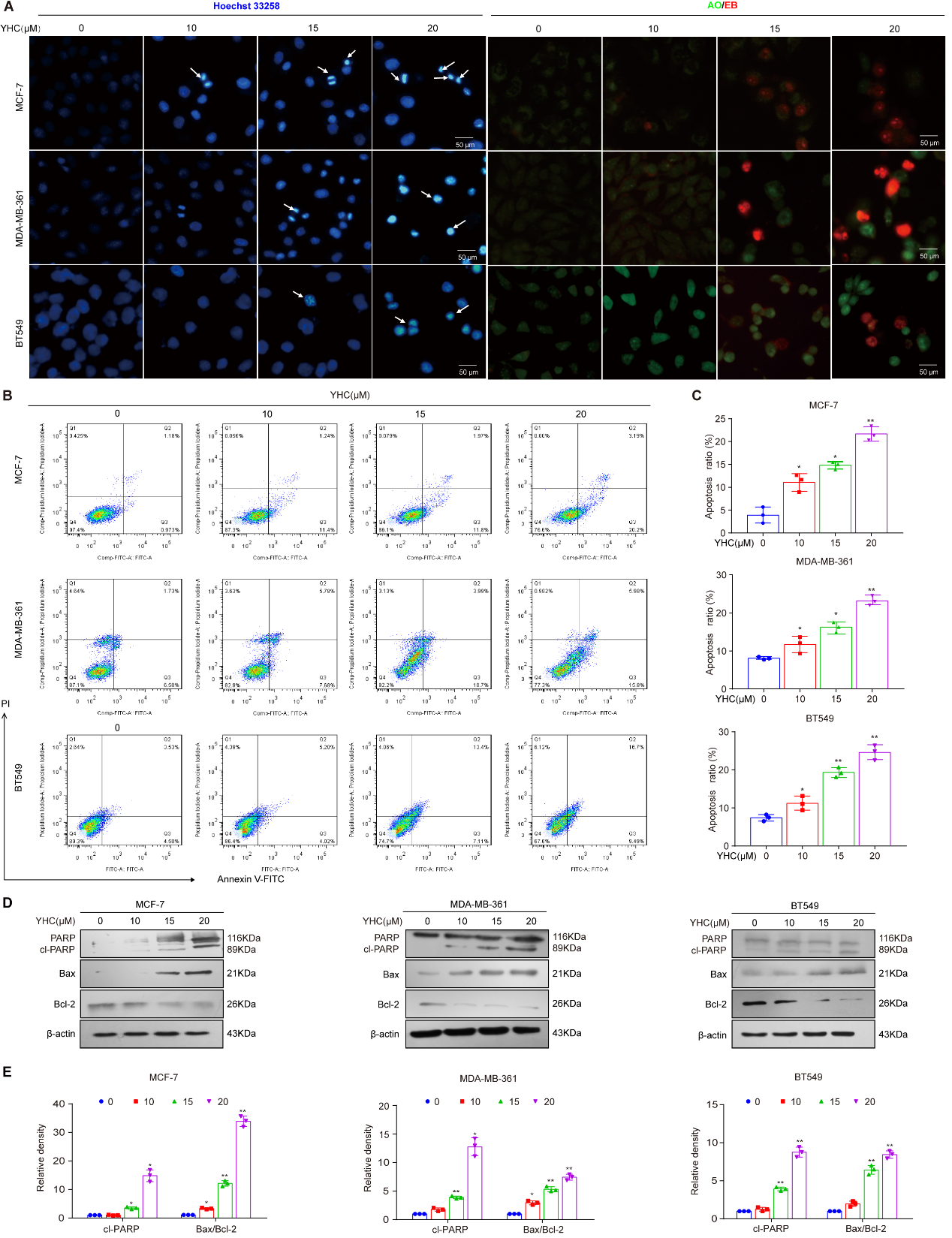


Figure S3 **Apoptosis induction contributed to the inhibitory effect of YHC on breast cancer cells.**

The cells were treated with different concentrations of YHC (0, 10, 15 and 20 μM) for 48 h. **A.** Apoptotic nuclear morphology was detected by Hoechst 33258 and AO/EB staining (Scale bar = 50 μm). Arrows indicated the apoptotic cells. **B, C.** Apoptotic ratio was determined by Annexin V-FITC/PI staining using flow cytometry. **D.** Expression levels of apoptosis-related proteins were detected by Western blot analysis. β-actin was used as a loading control. **E.** The band intensities were quantified by ImageJ software and the normalized ratio of PARP, Bax and Bcl-2 were calculated. The data were presented as the mean ± SD (n = 3). ^*^*P* < 0.05, ^**^*P* < 0.01, vs control group.


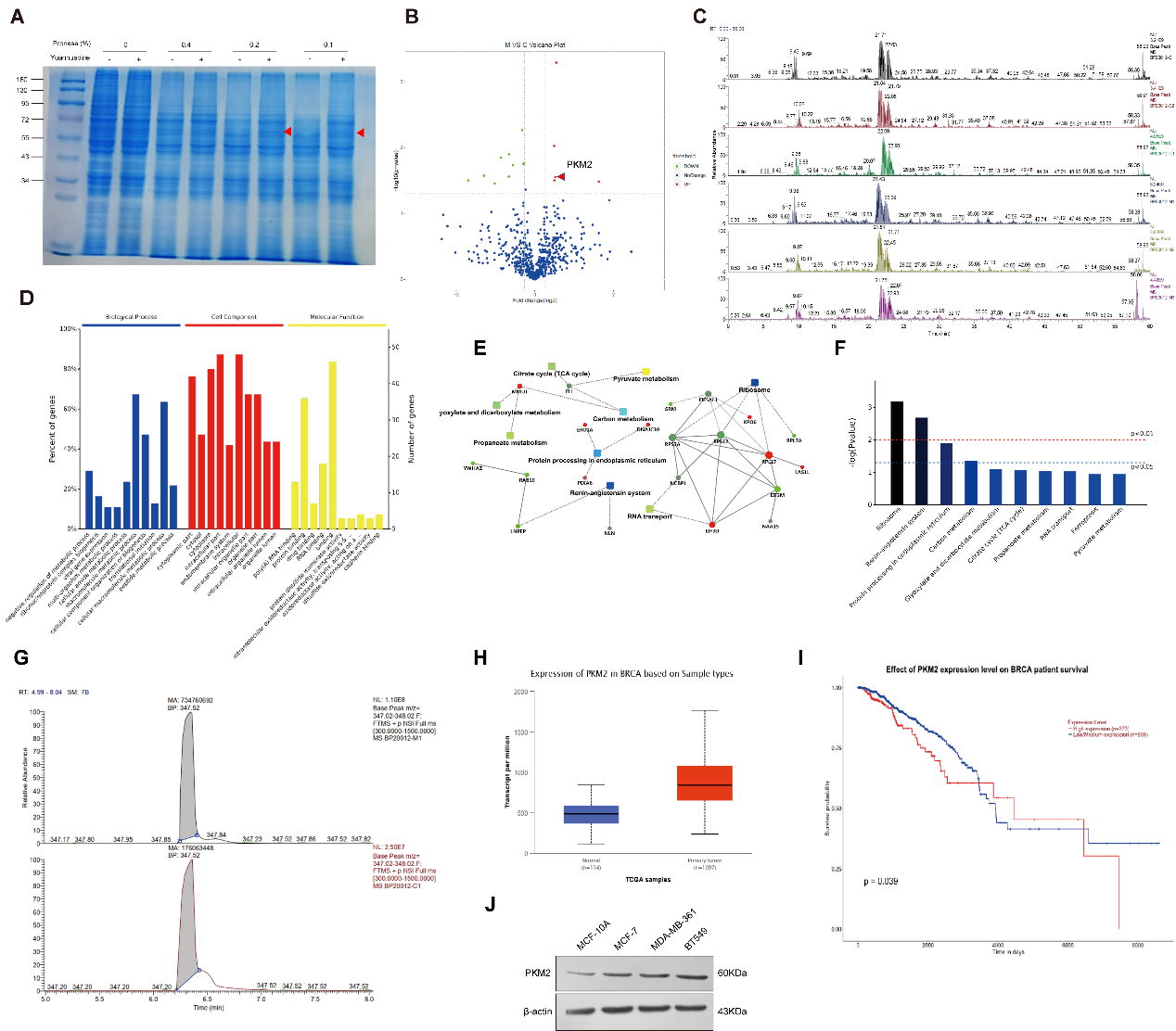


Figure S4 **Identification the potential target of YHC against breast cancer cells.**

**A.** Target identification of YHC using the DARTS/MS. **B.** Volcano plots showed the differentially expressed proteins in MCF-7 cells. **C**. MS confirmed the binding of YHC with proteins. **D**. GO enrichment analysis of the significantly upregulated and downregulated genes. **E**. PPI network for potential targets of YHC. **F**. KEGG analysis for potential targets of YHC. **G**. MS spectrum of a representative unique peptide of PKM2. **H.** Expression analysis of PKM2 in normal individuals and breast cancer patients (*P* < 0.01). **I.** Cumulative survival of breast cancer patients with low or high PKM2 expression (*P* = 0.039). **J**. Expression of PKM2 in breast cancer cells and normal breast cells. The data were presented as the mean ± SD (n = 3).


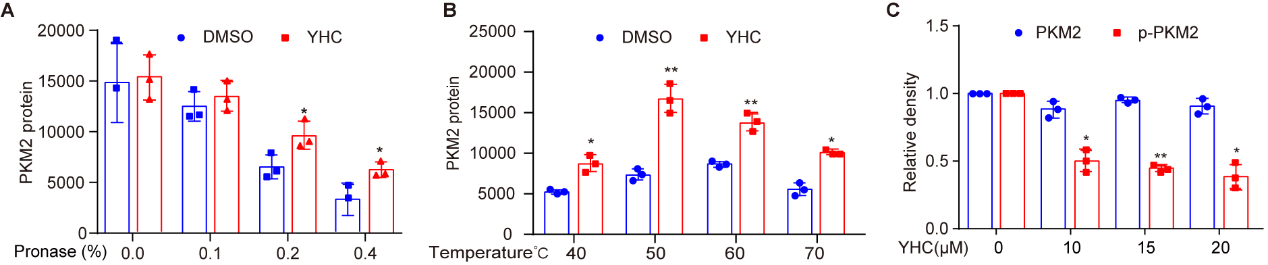


Figure S5 **A direct interaction between YHC and PKM2 was observed in breast cancer cells.**

A. Statistical histograms of the expression of PKM2 by DARTS in Fig. 2B. B. Statistical histograms of the expression of PKM2 by CETSA in Fig. 2C. C. Statistical histograms of the expression of PKM2 and p-PKM2 (Y105) by Western blot in Fig. 2F (YHC with 0, 10, 15 and 20 μM). The data were presented as the mean ± SD (n = 3). ^*^*P* < 0.05, ^**^*P* < 0.01, vs control group.


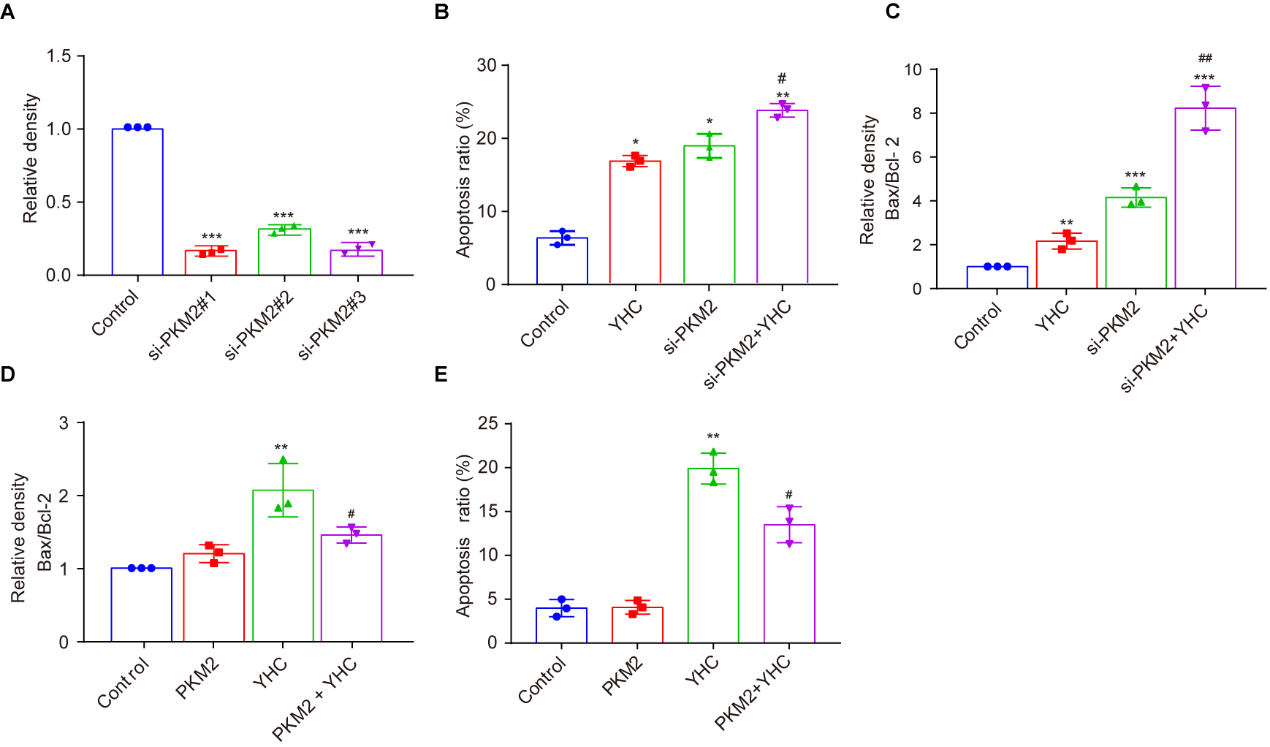


Figure S6 **YHC promoted apoptosis induction of breast cancer cells by targeting PKM2.**

A. Statistical histograms of the expression of PKM2 by Western blot in Fig. 2H. B. Statistical histograms of apoptotic ratios in Fig. 2I. C. Statistical histograms of the expression of Bax/Bcl-2 by Western blot in Fig. 2J. D. Statistical histograms of the expression of Bax/Bcl-2 by Western blot in Fig. 2K. E. Statistical histograms of apoptotic ratios in Fig. 2L. ^*^*P* < 0.05, ^**^*P* < 0.01, ^***^*P* < 0.001 vs control group. ^#^*P* < 0.05, ^##^*P* < 0.01 vs YHC treatment group.


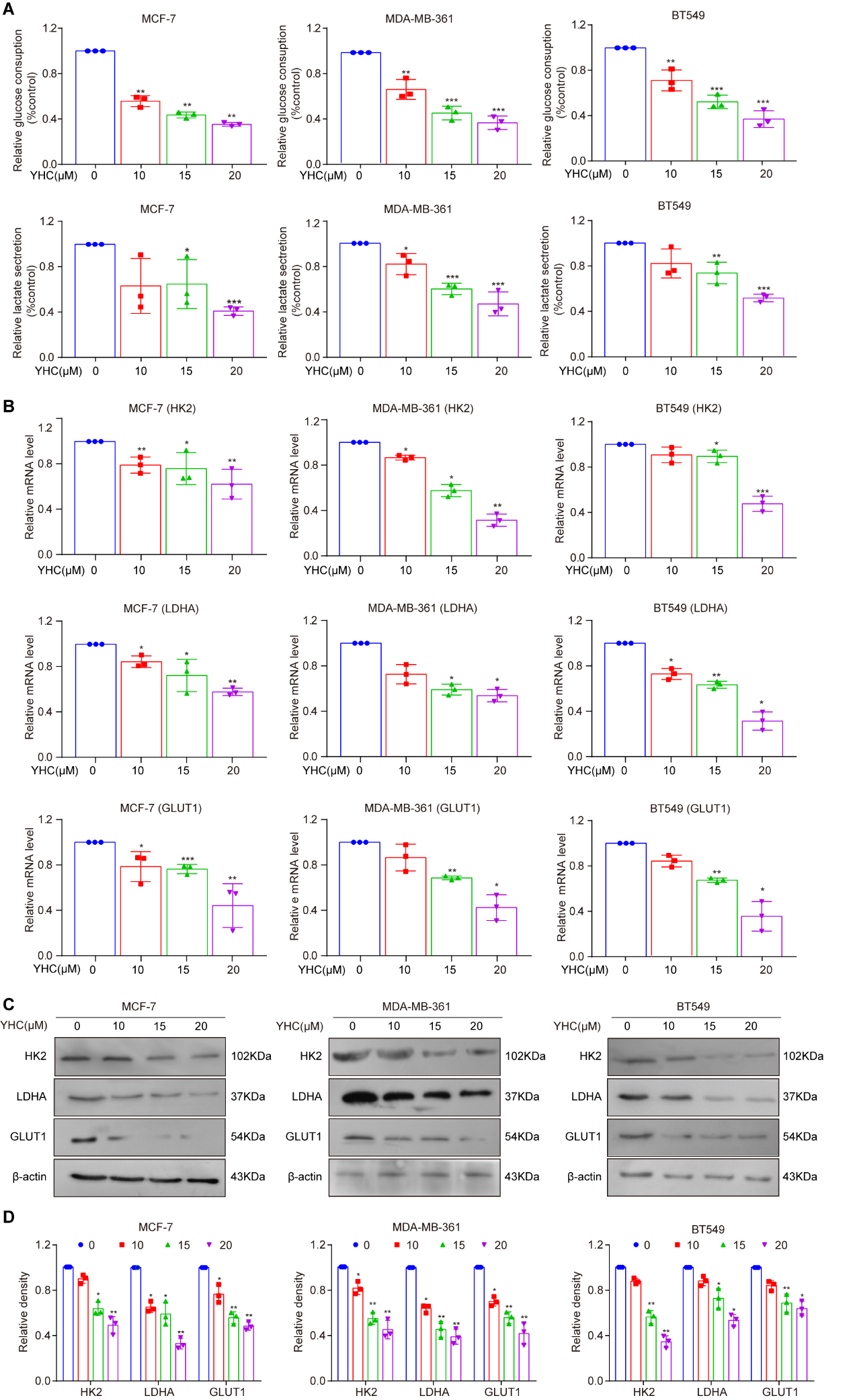


Figure S7 **The influence of YHC on the glycolytic pathway in breast cancer cells.**

The cells were treated with different concentrations of YHC (0, 10, 15 and 20 μM) for 48 h. **A.** The culture medium was collected after treatment with YHC to measure the glucose consumption and lactate production by glucose and lactate assay kit. **B.** The mRNA levels of HK2, GLUT1 and LDHA were detected by RT-PCR analysis. **C.** The expression levels of HK2, GLUT1 and LDHA were detected by Western blot. **D.** The band intensities were quantified by ImageJ software and the normalized ratio of HK2, LDHA and GLUT1 were calculated. The data were presented as the mean ± SD (n = 3). ^*^*P* < 0.05, ^**^*P* < 0.01, ^***^*P* < 0.001 vs control group.


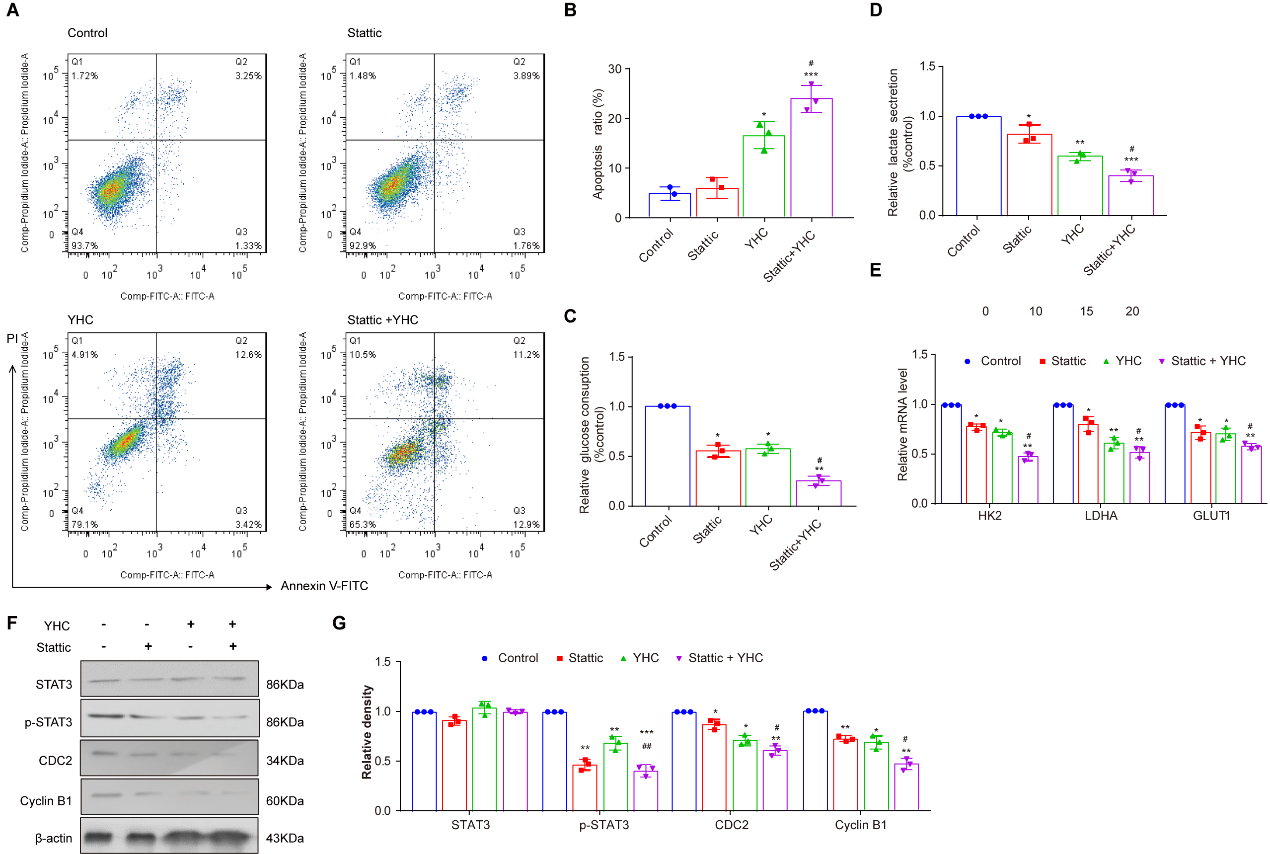


Figure S8 **YHC inhibited the growth of breast cancer cells by regulating STAT3 signaling pathway.**

In the absence or presence of Stattic for 1 h, cells were treated with YHC (15 μM) for 48 h. **A, B.** The Annexin V-FITC/PI staining assay was performed to analyze apoptosis using flow cytometry. **C, D.** The culture medium was collected to measure the glucose consumption and lactate production using glucose and lactate assay kit. **E.** The mRNA levels of HK2, GLUT1 and LDHA were detected by RT-PCR analysis. **F, G.** The expression levels of STAT3, p-STAT3 (Y705), CDC2 and Cyclin B1 were detected by Western blot analysis. The data were presented as the mean ± SD (n = 3). *^*^P* < 0.05, *^**^P* < 0.01, ^***^*P* < 0.001 vs control group; *^#^P* < 0.05, ^##^*P* < 0.01 vs YHC treatment group.


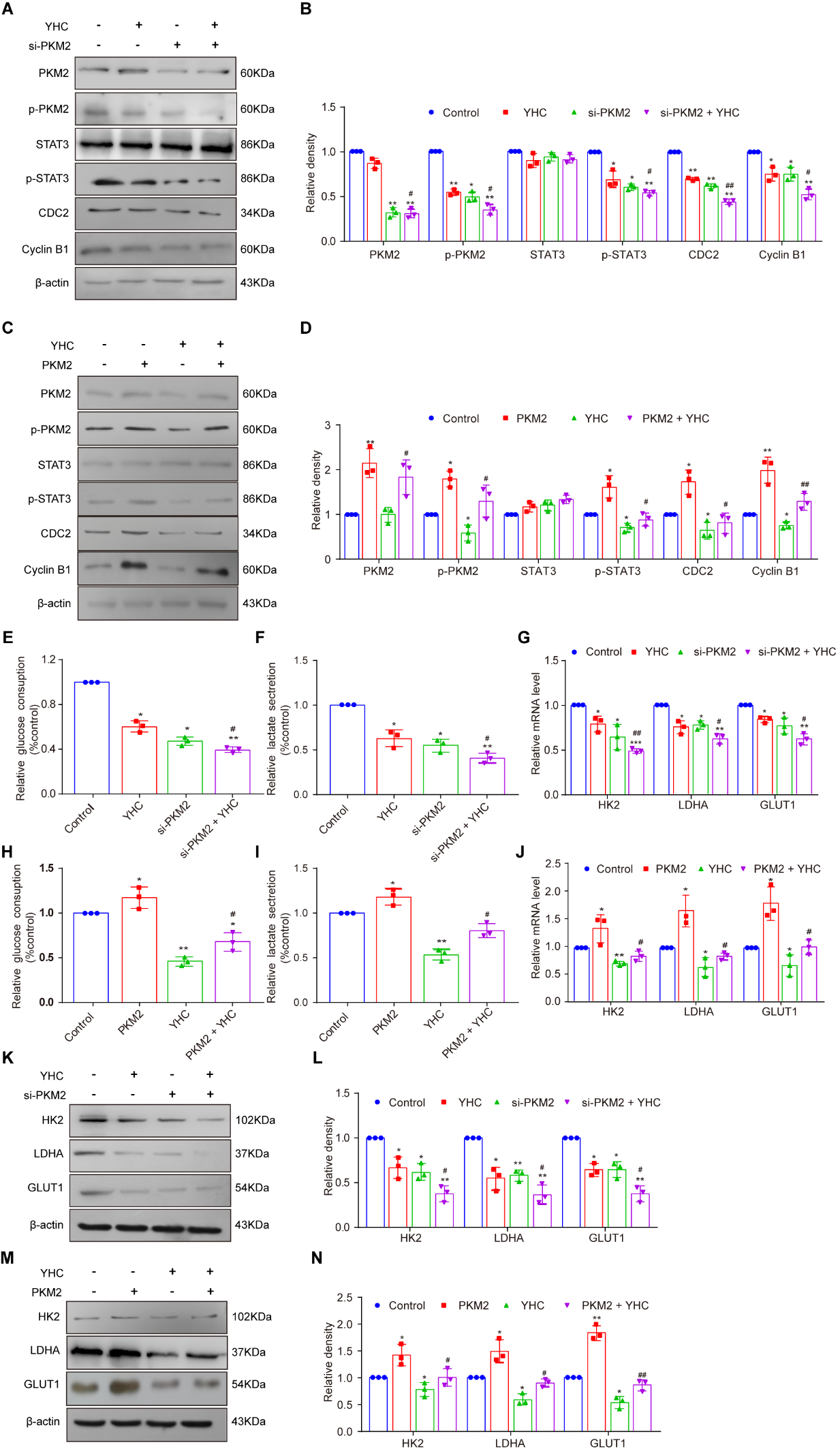


Figure S9 **YHC regulated STAT3 pathway and glycolytic pathway in breast cancer cells by targeting PKM2.**

The cells were treated with YHC (15 μM) for 48 h. **A-D.** The expression levels of PKM2, p-PKM2 (Y105), STAT3, p-STAT3 (Y705), CDC2 and Cyclin B1 in PKM2 siRNA-treated and PKM2-overexpressed MCF-7 cells were detected by Western blot. **E, F.** The culture medium was collected to measure the glucose consumption and lactate production in PKM2 siRNA-treated MCF-7 cells by glucose and lactate assay kit. **G.** The mRNA levels of HK2, LDHA and GLUT1 in PKM2 siRNA-treated MCF-7 cells were examined using RT-PCR. **H, I.** The culture medium was collected to measure the glucose consumption and lactate production in PKM2-overexpressed MCF-7 cells by glucose and lactate assay kit. **J.** The mRNA levels of HK2, LDHA and GLUT1 in PKM2-overexpressed MCF-7 cells were tested using RT-PCR. **K, L.** The expression levels of HK2, LDHA and GLUT1 in PKM2 siRNA-treated MCF-7 cells were evaluated by Western blot. **M, N.** Western blot assay was applied to detect the expression levels of HK2, LDHA and GLUT1 in PKM2-overexpressed MCF-7 cells. The data were presented as the mean ± SD (n = 3). ^*^*P* < 0.05, ^**^*P* < 0.01 vs control group; ^#^*P* < 0.05, ^##^*P* < 0.01 vs YHC treatment group.
