# Supplementary material for: A natural PKM2 targeting agent as a potential drug for breast cancer treatment
Source: Clin Transl Med. 2022 Dec 28;13(1):e1157. doi: 10.1002/ctm2.1157 (PMC9798039; doi:10.1002/ctm2.1157)
Supplement: Supplementary file 12 — Supporting information [file CTM2-13-e1157-s009.docx]

**Supporting Information 3:** Materials and methods

**Methods**

***Chemicals and reagents***

YHC (purity ≥ 98%) was isolated from *D. genkwa* in our laboratory. It was dissolved in dimethyl sulfoxide (DMSO) to make a stock solution. All cell lines used in this study were obtained from the American Type Culture Collection (ATCC). 3-(4,5-dimethylthiazol-2-yl)-2,5-diphenyltetrazolium bromide (MTT) was Sigma-Aldrich Co., Ltd (St. Louis, USA). Fetal bovine serum (FBS) was purchased from CLARK Bioscience. Dulbecco’s modified Eagle’s medium (DMEM), Phosphate balanced solution (PBS) and Antibiotics (100 U/mL penicillin, 100 μg/mL streptomycin) were purchased from HyClone, Inc (Utah, USA). PARP (sc-7150), Bcl-2 (sc-7382), Bax (sc-493), β-actin (sc-47778), CDC2 (sc-54), Cyclin B1 (sc-245) and horseradish-peroxidaseconjugated secondary antibodies (goat anti-rabbit or goat anti-mouse) were purchased from Santa Cruz Biotechnology (CA, USA). PKM2 (4053S), p-PKM2 (3827S), STAT3 (9139S, 12640S) and p-STAT3 (3145S) antibodies were purchased from Cell Signaling Technology (Massachusetts, USA). HK2 (66974-1-Ig), LDHA (19987-1-AP) and GLUT1 (66290-1-Ig) antibodies were purchased from Proteintech Group (Rosemont, USA). RIPA lysis buffer and BCA (bicinchoninic acid) assay kit was purchased from Beyotime (Shanghai, China). Immunocoprecipitation magnetic beads and Annexin V-FITC and propidium iodide (PI) were purchased from Bimake (Houston, USA). PVDF (polyvinylidene fluoride) membranes (0.2 μm) were purchased from Millipore (Massachusetts, USA). Glucose Assay Kit and Lactate Assay Kit were obtained from Jiancheng Biochemical Company (Nanjing, China).

***Cell culture***

MCF-7, MDA-MB-361, BT549 and MCF-10A were cultured in DMEM with 10% fetal bovine serum (FBS) and 1% penicillin/streptomycin at 37 °C in a humidified atmosphere with 5% CO_2_.

***Cell viability***

Cell viability was evaluated by MTT assay. After cells were treated with compounds for 48 h, 20 μL of MTT (5 mg/mL) was added for 4 h. Then 150 μL/well of DMSO was added to dissolve the crystals. The absorbance was measured at 490 nm using a microplate reader (Molecular Devices, Thermo, CA, USA).

***Colony formation***

Approximately 1000 cells/well were seeded into 6-well plates cultured for 10 days with indicated treatments to allow colony formation. The cells were fixed with 4% paraformaldehyde and stained with crystal violet. Then we counted colonies containing more than 50 cells.

***Hoechst 33258 staining***

The cells were incubated with YHC for 48 h and photographed using a fluorescence microscope after Hoechst 33258 staining. Hoechst 33258 staining was performed as described previously (Song et al., 2020).

***AO/EB staining***

The cells were incubated with YHC for 48 h and photographed using a fluorescence microscope after AO/EB staining at 37 °C. AO/EB staining was performed as described previously (Song et al., 2020).

***Cell apoptosis ratio detection***

Cells were seeded into 6-well plates (1.5 × 10^5^ cells/well) and incubated for 24 h to allow exponential growth, treated with YHC for 48 h. After incubation, the cells were washed twice in PBS and resuspended in an Annexin V-FITC/PI staining solution according to the manufacturer′s instruction. Apoptotic cells were immediately analyzed by flow cytometry. The samples were then analyzed with Flow Jo 7.6.5.

***Western blot analysis***

The total cellular samples were harvested and lysed in RIPA buffer and boiled for 10 min at 100 °C. Equal amount of protein (30 μg) were separated on a 10% SDS-PAGE gel and transferred to nitrocellulose membranes. The membranes were blocked with 5% BSA and probed with a primary antibody (1:1000) followed by the corresponding secondary antibody (1:5000). Immunoreactive bands were visualized with a chemiluminescence kit followed by incubation with HRP-conjugated secondary antibodies. The density of protein bands was calculated by the ImageJ software.

***DARTS/MS***

Cell lysates were obtained from MCF-7 cells. Cells were scraped and lysed with NP-40 lysis buffer. After being centrifuged for 15 min at 16,000 g, the supernatant was obtained and protein content was quantified using BCA solution. Samples were treated with the drugs YHC and DMSO for 2 h at room temperature. Samples were then incubated with pronase, as indicated, for 1 h at room temperature. The pronase digestion was ceased by adding SDS-PAGE sample loading buffer and heated at 95 °C for 5 min. The protein samples were run on a 10% Bis-tris gel and stained using Coomassie blue. The gel with significant change was further analyzed by MS analysis. The experimental data containing the peptide mass (m/z) values were analyzed using MASCOT peptide mass fingerprint.

***Molecular docking***

The X-ray crystal structure of the PKM2 (PDB: 6NU5) and a resolution of 1.60Ǻ was obtained from the RCSB protein data bank (PDB) (http://www.wwpdb.org). The protein was optimized by the iGEMDOCK software to add all hydrogen atoms and remove water molecules. The structural relation was carried out for the docking calculations using the AUTODOCK 4.2 SUITE of program. The visualization of resulting complex geometry was performed on Discovery Studio 4.5 program.

***UALCAN analysis***

UALCAN database (http://ualcan.path.uab.edu) contains 31 types of cancer patients with clinical and RNAseq data (Wang et al., 2020; Xi et al., 2019). UALCAN is an interactive portal, which can deeply analyze the relationship between the expression level of target genes in TCGA and the clinical data of patients. In this study, the expression level of PKM2 in normal individuals and breast cancer patients was analyzed.

***The cellular thermal shift assay (CETSA)***

MCF-7 cells were harvested and washed with PBS, and then diluted in lysis buffer. The soluble fraction (lysate) was extracted by ultrasonic and separated from the cell debris by centrifugation at 16,000 g for 15 min at 4 ℃. For the thermal aggregation curve experiments cell lysates were diluted with lysis buffer and divided into two aliquots, one aliquot was treated with YHC and the other aliquot was treated with DMSO (control). After 60 min incubation at room temperature, the respective lysates were divided into smaller (50 μL) aliquots in 0.2 mL tubes and then heated at designated temperatures ranging from 40 to 70 ℃ individually at different temperatures for 3 min. Appropriate temperatures were determined in preliminary CETSA experiments. After this 3-min room temperature incubation, the samples were immediately transferred to ice. The heated lysates were centrifuged at 16,000 g for 15 min at 4 ℃ in order to separate the soluble fractions from precipitates. Soluble proteins in the supernatants were transferred into new 0.2 mL microtubes and then analyzed the expression of PKM2 or STAT3 by immunoblot analysis.

***Surface plasmon resonance assay***

The interaction between small molecule and protein was evaluated by surface plasmon resonance (SPR) using a Biacore T100 instrument (GE Healthcare, USA). In brief, the specific interaction of samples with the immobilized PKM2 was assessed. All samples were analyzed at a flow rate of 30 μL/min with running buffer (PBSP + 5% DMSO) and contact time of 300 s. The binding of YHC to immobilized PKM2 was monitored by applying YHC (0.2-50 μM) in 1 × PBS (PH 7.4). The equilibrium dissociation constant (K_d_) was obtained using BIA evaluation software.

***RT-PCR***

Total RNA was extracted using Trizol reagent (TaKaRa, Shiga, Japan). cDNA synthesis was performed using a Revert Aid First Strand cDNA Synthesis kit (TaKaRa, Shiga, Japan). Subsequently, the synthesized cDNA was amplified. The following primers were used: PKM2: forward 5′-CTATGGGAACAGAGGGTCTTTT-3′ and reverse 5′-TGGTTTACTCCATTCCACAAGA-3′, STAT3: forward and reverse HK2: forward 5′-CGACAGCATCATTGTTAAGGAG-3′ and reverse 5′-GCAGGAAAGACACATCACATTT-3′, GLUT1: forward 5′-TGTCTGGCATCAACGCTGTCTTC-3′ and reverse 5′-CCTGCTCGCTCCACCACAAA-3′, LDHA: forward 5′-AGGTGATCAAACTCAAAGGCTA-3′ and reverse 5′-CCCAAAATGCAAGGAACACTAA-3′.

***Coimmunoprecipitation analysis***

Immunoprecipitation assay was conducted by using the protein A/G magnetic beads according to the manufacturer′s instructions. MCF-7 cells were then lysed using lysis buffer. The pre-clear lysate was added to the control agarose resin to avoid nonspecific interactions with the resin matrix. Next, the coimmunoprecipitation process was performed by incubating treated lysate with antibody immobilized coupling resin, the quenched antibody coupling resin as a negative control at the same time. Finally, the resin was heated at 100 °C for 10 min with SDS sample buffer to prepare SDS-PAGE analysis, and the target protein was detected by Western blot.

***Glucose consumption***

Control and YHC-treated cells were cultured for 48 h. The cultured medium was harvested. The concentrations of glucose were determined by the glucose assay kit according to the manufacturer′s instructions.

***Lactate measurements***

Control and treatment with YHC cells were cultured for 48 h. The cultured medium was harvested. The concentrations of lactate were determined by the lactate assay kit according to the manufacturer′s instructions.

***Transfection of siRNA***

Cells were transfected and cultured for another 24 h before subsequent experiments. MCF-7 cells were transfected with negative control siRNA (si-NC) or siRNA targeting PKM2 (si-PKM2), using Lipofectamine 2000 following the manufacturer′s instructions. The specific sequences used were as follows: si-PKM2#1, sense 5′-CCGGAUACUUACAGAUGUATT-3′, si-PKM2#2, sense 5′-CCGGAUACUUACAGAUGUATT-3′, si-PKM2#3, sense 5′-CCGGAUACUUACAGAUGUATT-3′. Then cells treated with YHC, were examined for subsequent experiments.

***Plasmid constructs for overexpression***

Cells were seeded and transfected at 70-80% confluency with PKM2 (untagged)-Human plasmid (Genechem, Shanghai, China) using Lipofectamine 2000 reagent (Invitrogen, Shanghai, China). After the indicated treatments, transfected cells were harvested for the followed analysis.

***In vivo anti-tumor activity***

BALB/c nude mice at the age of six weeks were obtained from the Liaoning Changsheng Biotechnology Co., Ltd. (Shenyang, China). All experiments were performed in a specific pathogen-free unit of Liaoning Changsheng Biotechnology Co., Ltd. This study was approved by the Ethics Committee for Animal Experiments of Liaoning Changsheng Biotechnology Co., Ltd. (CSE20201113). MCF-7 cells (1 × 10^5^ cells) were implanted subcutaneously into the right backsides of each nude mouse. After 8 days, when the tumor size reached about 100 mm^3^, the mice were randomly divided into three groups (n = 5) and intraperitoneally injected with vehicle control, YHC (0.25 and 0.5 mg/kg body weight) or Dox (0.25 mg/kg body weight) every two days for two weeks. The body weights and tumor sizes were measured every day, and the tumor volumes were determined by caliper measurements and calculated by the formula: 0.5236 × L × W^2^, where L was the long axis and W was the short axis of the tumor. At two weeks, the mice were euthanized by isflurane and the tumors, hearts, livers, spleens, lungs and kidneys were excised immediately to evaluate the anti-tumor activity and toxicity of YHC.

***H&E Staining and Immunohistochemistry***

Mouse major organs (heart, liver, spleen, lung, kidney) and tumors were isolated and fixed in 10% buffered formalin for 3 days and embedded in paraffin. The tissues were sectioned at a thickness of 4 μm, and the obtained organ sections were prepared orderly by dewaxing, staining in hematoxylin and eosin-phloxine solution, then dehydrated and mounted with neutral resin. Subsequently, the tumor paraffin sections were deparaffinized, rehydrated, and subjected to antigen retrieval. Immunostaining was performed by the streptavidin-peroxidase (S-P) method. Finally, the sections tissue morphology were observed using an OLYMPUS microscope.

***Statistical analysis***

All experiments were repeated at least three times and the data were presented as mean ± SD. Statistical comparisons among the different groups were made using Student′*t*-test and one-way ANOVA to analyze the significance of the difference between two groups or among multiple groups, respectively in GraphPad Prism (Version 7.0, GraphPad Software). *P* -values < 0.05 were considered statistically significant.
